# The use of an augmented reality app to support an exercise intervention for children and young people with cancer: perspectives of users and exercise and healthcare professionals in the FORTEe trial

**DOI:** 10.3389/fped.2026.1743212

**Published:** 2026-03-09

**Authors:** Hayley Marriott, Alba Solera-Sanchez, Stanley Windsor, Kim Straun, Marie A. Neu, Elias Dreismickenbecker, Francesca Lanfranconi, Emanuele Villa, Joachim Wiskemann, Nikolai Bauer, Miriam Götte, Ronja Beller, Filippo Spreafico, William Zardo, Peter Wright, Joerg Faber, Eila Watson

**Affiliations:** 1 School of Sport, Nutrition and Allied Health Professions, Faculty of Health Science, and Technology, Oxford Brookes University, Oxford, United Kingdom; 2 School for Policy Studies, University of Bristol, Bristol, United Kingdom; 3 University Medical Center of the Johannes Gutenberg-University Mainz, Childhood Cancer Center Mainz, Mainz, Germany; 4 Fondazione Monza e Brianza per Il Bambino e La Sua Mamma, Monza, Italy; 5 Exercise Oncology Research Group, Department of Medical Oncology, Heidelberg University Hospital, Medical Faculty Heidelberg, Heidelberg University, Heidelberg, Germany; National Center for Tumor Diseases (NCT), NCT Heidelberg, a partnership between DKFZ and the Heidelberg University Hospital, Heidelberg, Germany; 6 University Hospital Essen, West German Cancer Center, Essen, Germany; 7 Clinics for Paediatrics III, Department of Paediatric Haematology/Oncology, West German Cancer Centre, University Hospital Essen, Essen, Germany; 8 Oncology Unit, IRCCS Istituto Giannina Gaslini, Genoa, Italy; 9 Pediatric Oncology Unit, Fondazione IRCCS Istituto Nazionale dei Tumori, Milan, Italy; 10 Oxford Institute of Applied Health Research, Faculty of Health Science, and Technology, Oxford Brookes University, Oxford, United Kingdom

**Keywords:** augmented reality, childhood cancer, exercise, exercise oncology, smartphone app

## Abstract

**Background:**

Mobile health (mHealth) technologies are increasingly used in paediatric oncology to promote physical activity, with growing evidence supporting their feasibility and effectiveness. Augmented reality (AR) is emerging as a promising addition, offering interactive features that may enhance participation in exercise for young people. As mHealth tools evolve, understanding user experiences and implementation challenges is essential to inform wider adoption in healthcare. The FORTEe clinical trial evaluates an individualised exercise programme designed for children, adolescents and young adults with cancer (CAYA). This sub-study explores the perspectives of CAYA and exercise and healthcare professionals involved in delivering the exercise intervention (exercise professionals) on the use of a novel AR application (app), designed to facilitate home-based exercise as part of the broader intervention. Key app features include personalised exercise programmes, AR demonstrations using a child-like avatar, and an integrated exercise diary.

**Methods:**

CAYA (9–21 years), and exercise professionals from the FORTEe trial's technology sub-study (six centres) were eligible. To explore experiences and perceptions of the AR app, half-structured interviews were conducted with CAYA and an anonymous online survey administered to exercise professionals. Interview data and open-ended text from survey was analysed using inductive qualitative content analysis. Survey data was analysed using descriptive statistics.

**Results:**

A total of 46 CAYA (mean age 13.6 ± 2.7 years, 39% female) provided feedback on the AR app via interviews, and 31 exercise professionals completed the survey. CAYA and exercise professionals reported generally positive experiences, finding the AR demonstrations novel and engaging. The personalised workouts were valued by both groups. However, both groups reported some technical difficulties that impacted reliability, and that some features, such as the exercise diary, lacked usability. Exercise professionals emphasised the app should complement rather than replace face-to-face sessions. To enhance the app further, both groups suggested incorporating gamification and avatar customisation.

**Conclusion:**

This study emphasises the potential of AR technology to increase engagement in exercise among CAYA and highlights ways to optimise the technology. While AR shows promise in paediatric oncology care, it should complement in-person exercise interventions. Future development should prioritise user-friendly design, personalised approaches, and equal access for young patients.

## Introduction

Mobile health (mHealth), a subset of electronic health (eHealth), is described as a class of health technologies, specifically applications that are delivered through mobile and often internet-connected communication devices. One of the main characteristics of mHealth is the delivery through devices that are “tethered” to the user such as smartphones and smartwatches (1). The benefits of mHealth interventions, such as their accessibility, cost-effectiveness, ability to personalise and tailor content, and the capacity to deliver real-time strategies to users, have likely contributed to the noticeable increase in their prevalence (2). mHealth has been identified as a promising tool to promote self-management in long-term health conditions in children and adolescents (3). Exercise interventions with mHealth components are emerging in paediatric oncology, offering tailored exercise programmes that meet participants' unique health needs and demonstrating both feasibility and effectiveness (4). mHealth interventional research has shown an enhanced adherence to exercise programmes (5), improved symptom monitoring and management (6, 7), body mass index (BMI) scores (4), physical fitness and body composition (8) and positive behaviour change (9). Findings suggest that integrating mHealth tools into standard care has been well-received by children and young adults with cancer (CAYA) (10), indicating their potential as effective adjuncts to standard cancer care pathways.

These developments position mHealth as a promising but incomplete solution, particularly for complex, engagement-dependent interventions such as exercise in paediatric oncology. While healthcare professionals often recognise the potential benefits of digital tools, concerns have been raised regarding their impact on patient-clinician relationships and long-term sustainability (11). Barriers related to infrastructure, training, and perceived clinical necessity further underscore that facilitators and challenges may vary across different mHealth tools (11). For these reasons, alongside evaluating feasibility and acceptability among paediatric users, it is critical to consider the perspectives of professionals delivering mHealth-supported interventions.

Within the mHealth context more immersive and interactive technologies have attracted growing interest. Among these, augmented reality (AR) and virtual reality (VR) are emerging as potentially transformative technologies in healthcare (12). VR involves the use of a specialised headset to simulate an environment in which the user is fully immersed (12), whereas AR overlays digital information in the physical environment, enhancing real-world perception and interaction (13). In paediatric oncology VR has shown promise, with evidence indicating its potential to reduce procedure-related anxiety and pain through immersive distraction, particularly when used alongside caregiver involvement (14). AR, by contrast, overlays digital elements onto real-world environments rather than fully immersing the user in a virtual one (15). The differences may be particularly relevant for exercise-based interventions as AR allows users to interact with their physical surroundings while engaging in digital content, whereas VR is associated with higher rates of cybersickness, including nausea and dizziness (16). Both AR and VR offer unique affordances and challenges for exercise interventions and considerations such as tolerability, treatment-related side effects and type of exercise must be considered when designing interventions.

While general AR applications, such as Pokémon GO, are popular among young people, no AR apps, to date, have been designed to tailor exercise programmes specifically for CAYA undergoing cancer treatment. To fill this gap, an interdisciplinary team of software developers, digital health academics, and exercise professionals collaborated to create an AR smartphone application suitable for CAYA with cancer aged 9–21 years old (17). The AR application has been designed to use as a supplementary technology alongside an individualised 8–10 week personalised exercise programme, as part of the FORTEe clinical trial (18). The FORTEe trial is a large cross-Europe multi-centre, randomised clinical trial, investigating the effects of a personalised exercise intervention in children and young people undergoing cancer treatment. The purpose of this study is to explore the perspectives of both CAYA with cancer and professionals involved in delivering the exercise intervention (hereafter exercise professionals) regarding use of the novel AR app delivered as part of the broader FORTEe clinical trial.

## Methods

### Overview

This technology sub-study of the FORTEe trial was conducted in six of the 10 trial centres, including one UK centre, two Italian centres, and three German centres (Appendix A). This sub-study was designed as a qualitative evaluation of user perspectives, complemented by supplementary descriptive survey data from professionals involved in delivering the exercise intervention.

### FORTEe trial design

The FORTEe trial protocol is detailed elsewhere (18) was conducted in 10 centres across 7 countries in Europe. The study is examining the effects of an individualised exercise programme in CAYA (aged 4–21) receiving cancer treatment. The FORTEe study, including this sub-study, was approved by the Ethics Committee of the Medical Chamber of Rhineland-Palatinate (Ref No: 2021- 15904) as well as the local ethic committees of all FORTEe trial sites. Trial participants were randomly assigned to one of two groups: the exercise group, offered an individualised exercise programme over a period of 8–10 weeks, and the control group, which received usual care. The exercise intervention consisted of face-to-face supervised sessions and optional remote supervised sessions.

### FORTEe AR app

**Figure 1:**
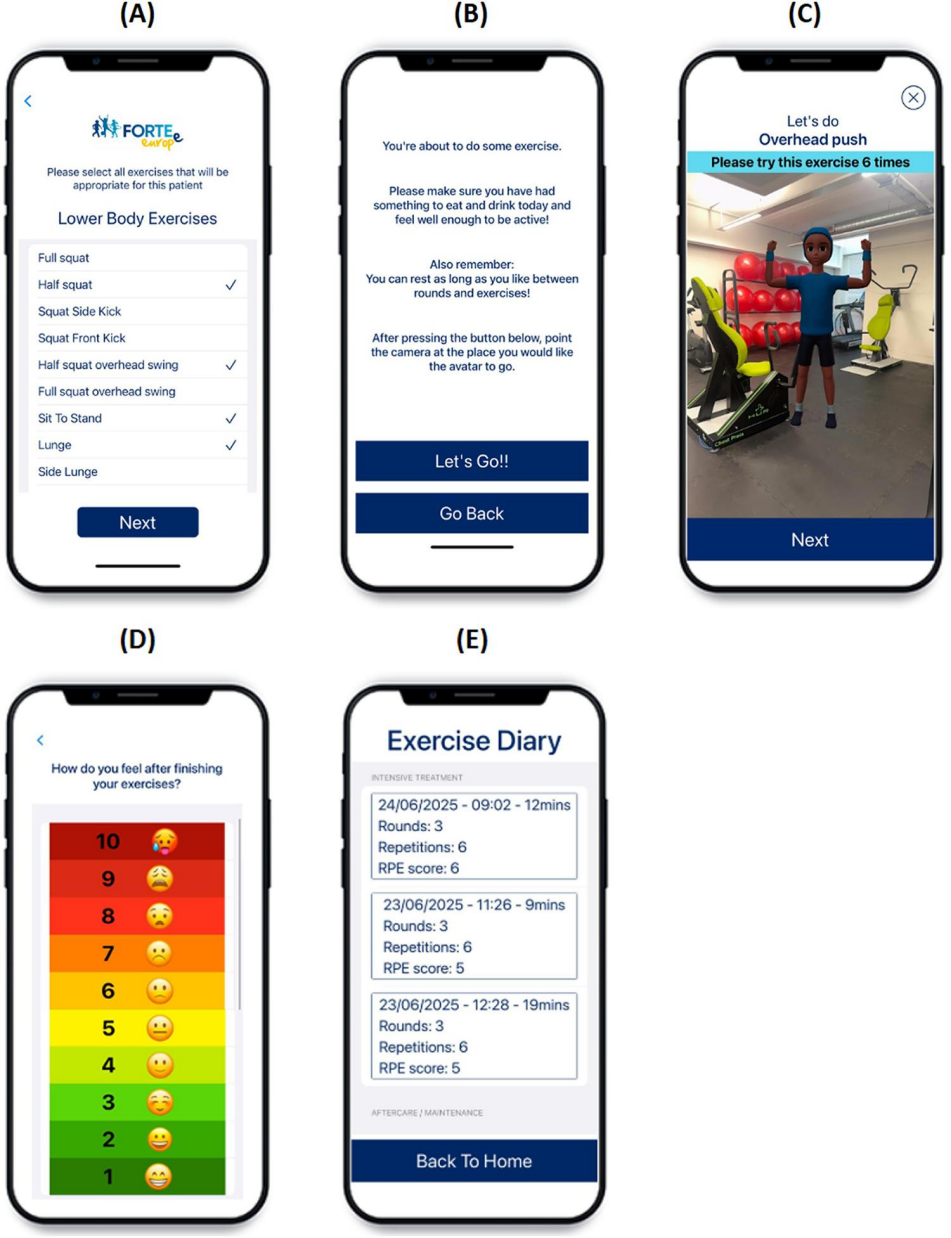
AR app screens **(A)** exercise selection, **(B)** Pre-exercise screen, **(C)** exercise session, **(D)** RPE scale, **(E)** exercise diary.

The AR app was developed for the FORTEe clinical trial to enable participants to perform strength and mobility exercises with animated avatar demonstrations using AR technology. It was specifically designed to enhance engagement, particularly outside clinical settings, by delivering personalised workouts tailored to participants diverse health and fitness needs. The app operates entirely offline (both use and data collection), ensuring privacy-by-design. The app was developed with two settings: intensive treatment phase and aftercare. As the majority of study participants used the app in the intensive treatment phase, this paper will focus on the “intensive” setting of the app. To personalise the app, an exercise professional was required to select exercises from a predefined list of 58 exercises, including seated variations, categorised into lower body, upper body, and core exercises. Each exercise session consisted of a circuit of six exercises randomly selected from the pre-selected exercise list. The exercise professional selected the number of repetitions (1–6) (the number of times to perform a single exercise), and sets (1–3) (a group of consecutive repetitions completed without rest) to create an individualised exercise programme. Following a supervised familiarisation session, designed to ensure participants understood the app's functionality and exercise form, participants were encouraged to use the AR app independently. Participants were guided through the exercises by AR avatar demonstrations. The avatar was designed to be child-like in appearance to enhance engagement. A usability study was conducted with age-matched children, and their feedback was incorporated into subsequent iterations of the app design (Marriott et al., 2026 *under review)*. Once the exercise session was completed, the participant completed a rate of perceived exertion (RPE) scale, and the completed exercise session was automatically recorded in the app's exercise diary. Figure 1 shows a selection of example screens from the app interface. In the intensive setting of the app, progression required the exercise professional to re-select exercises and adjust the number of repetitions and sets. As the app operates offline, all adjustments were made in person.

#### Recruitment

FORTEe participants aged 9–21 years enrolled at the six centres were invited to use the AR app. Those who agreed were provided with a study iPhone, tripod, charging leads to borrow, and written instructions to take home. Centres only had a limited number of study iPhones to lend to participating CAYA. Familiarisation session(s) with each participant were conducted prior to use with a FORTEe exercise professional. Participants in the intervention group were invited to use the AR app during the intervention phase and beyond, whilst the control group were invited to use the AR app only once the intervention period had concluded. While participants were encouraged by the study exercise professionals to use the app, participation was optional, and no specific frequency of use was mandated.

#### Data collection

**Figure 2:**
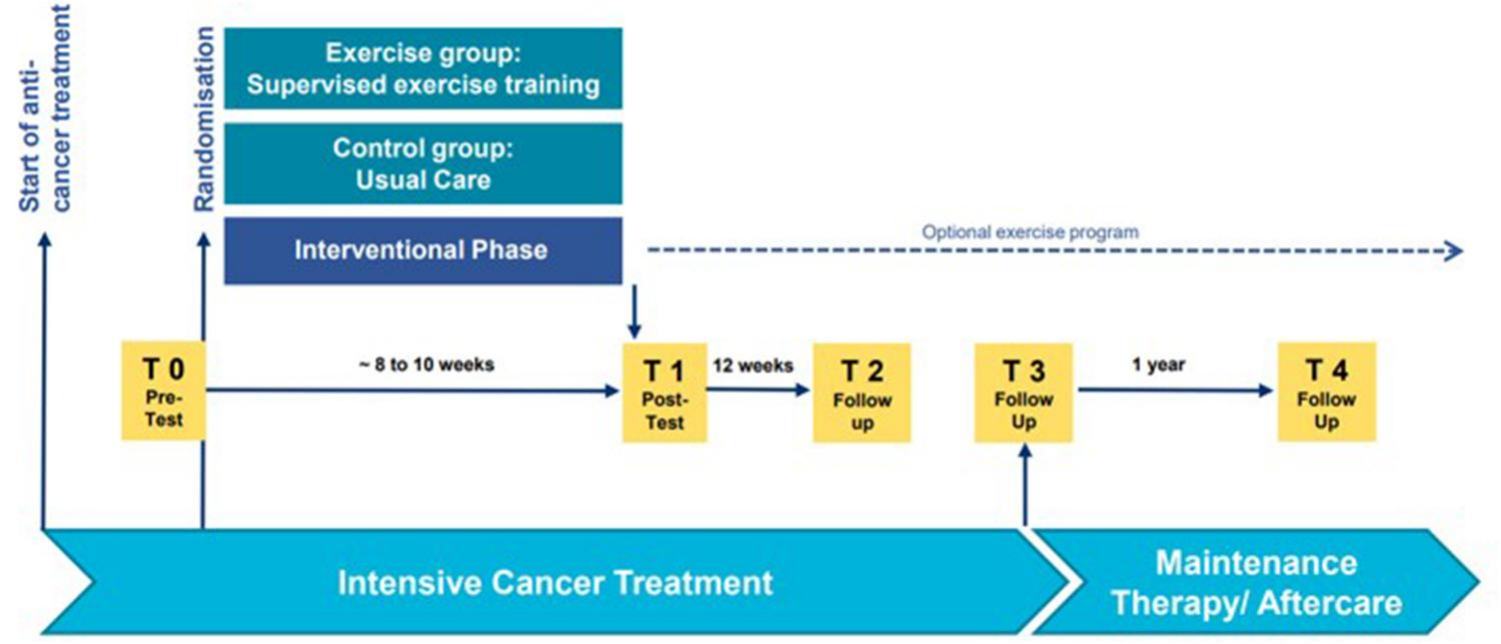
Data collection timeline (Neu et al. 2025).

All study participants were invited to participate in a half-structured (semi-structured) interview at multiple time points throughout the FORTE trial: baseline (soon after diagnosis) (T0), post intervention (T1), and at three follow up assessments (T2-T4) (see Figure 2). Interviews were conducted by a member of the local FORTEe team, either face to face or over the telephone. During the interviews, participants in the intervention group were asked about their use of, and experiences of the AR app at T1-T4, while those in the control group were asked about the app at T2-T4. The interviewer followed a set of questions with example prompts (see Appendix B) which covered participant perspectives on app design, app interface, exercise demonstrations, exercise selection, AR technology and the equipment provided. Interviews were conducted in the native language of the respective centre. Interviews were not audio-recorded, but responses were written down by the interviewer and translation to English was then undertaken (by researchers within each centre).

Exercise professionals affiliated with FORTEe centres were invited to complete an anonymous, web-based survey. Invitations were distributed via internal project communication channels. The survey was administered towards the end of the overall data collection phase of the FORTEe trial. The survey tool, developed for this sub-study, comprised closed (*n* = 12), and open-ended (*n* = 13) items designed to explore key implementation components. Topics included perceptions of the onboarding process, experiences training other staff, conducting familiarisation sessions with study participants, and the integration of the AR app as a supplementary training tool during supervised exercise sessions. Closed-ended survey questions used five-point Likert scale responses (e.g., from “strongly disagree” to “strongly agree”). The survey tool is available from the authors on request.

#### Data analysis

Written records from the CAYA interviews and open-ended text from the EHCP survey were analysed separately using an inductive content analysis approach (19, 20) Initial coding of the responses was conducted independently by two researchers (HM and AS) using an inductive approach. Following discussion (HM, AS and EW) a coding framework was agreed and applied to the data. Codes were then grouped into categories which summarised key patterns across the dataset. The analysis was descriptive in nature and aimed to systematically capture and structure participant perspectives rather than to generate theory.

For the survey data, descriptive statistics, including percentages, were used to summarise exercise professionals responses to each item. No inferential statistics were applied due to the exploratory nature of the survey.

## Results

**Figure 3:**
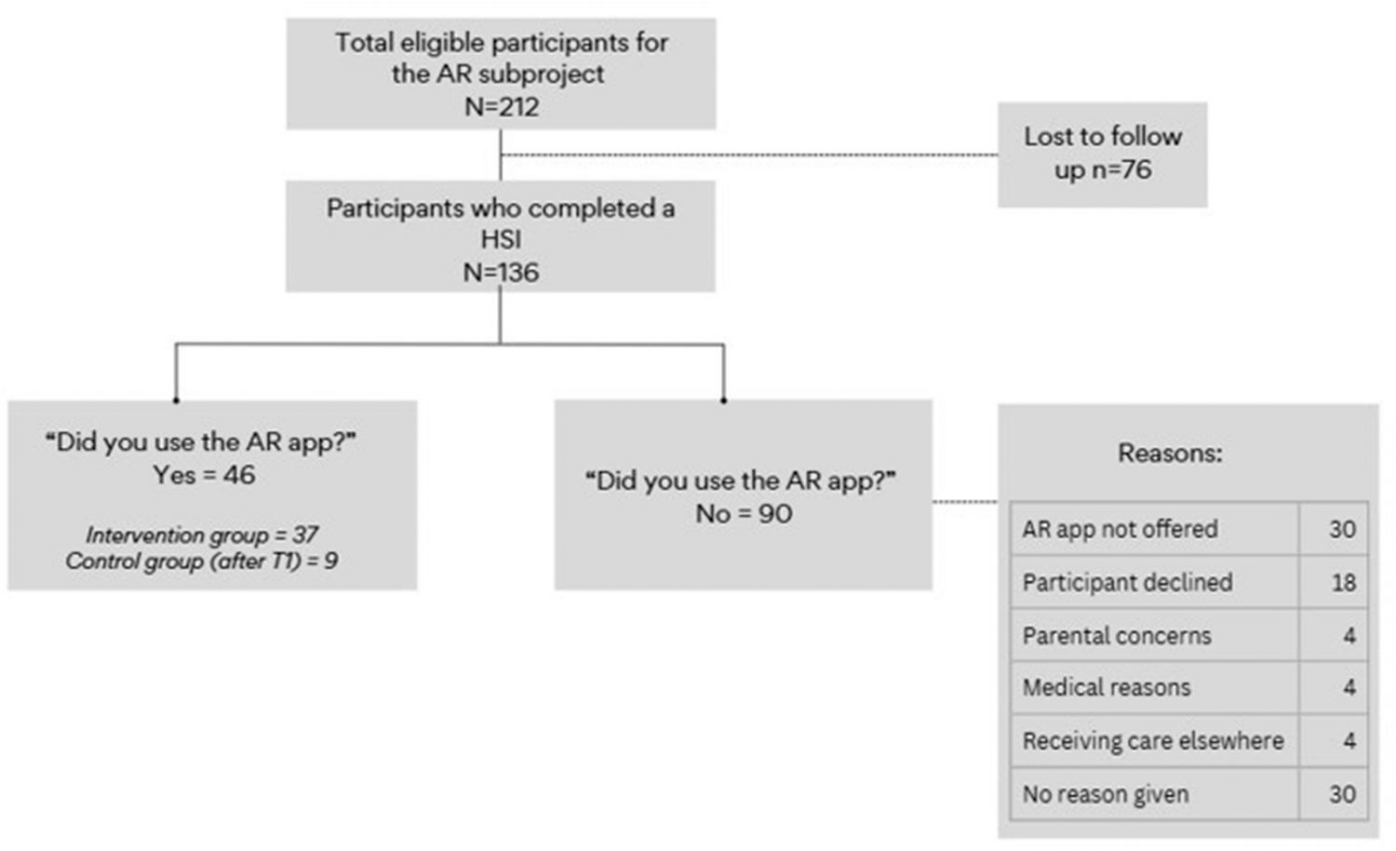
Flowchart of participant inclusion. HSI, half-structured interview; AR, augmented reality.

Figure 3 illustrates the inclusion of FORTEe participants eligible for the AR subproject.

Table 1 presents the demographic and clinical characteristics of CAYA who provided feedback on the app.

**Table 1:** CAYA demographic and clinical characteristics (*N* = 46).

Participant characteristic	Value
Age, years ± SD (age range)	13.6 ± 2.7 (9–19 years)
Sex (*n*, %)	
Male	28 (61%)
Female	18 (39%)
Diagnosis (*n*, %)	
Leukaemia	19 (41%)
Lymphoma	13 (28%)
Malignant bone tumours	5 (11%)
Soft tissue and other extraosseous sarcomas	4 (9%)
Neuroblastoma	2 (4%)
Renal tumours	1 (2%)
Germ cell tumours	1 (2%)
Other	1 (2%)

CAYA: children, adolescents and young adults with cancer.

### Qualitative findings

Experiences are grouped by themes and drawn from 46 CAYA who shared their perspectives during the half-structured interviews (35 responses at T1, 20 at T2, 5 at T3 and 6 at T4), along with open-ended text comments from the exercise professionals survey. Although the CAYA data was initially analysed by time point and intervention arm, no clear differences emerged either longitudinally or between the intervention and control groups. The findings are therefore presented collectively in the sections that follow. Additionally, 31 exercise professionals, from the FORTEe consortium, provided their feedback on the AR app in a series of open-ended questions. Survey responses indicated that these professionals had a range of experience with the app, including app set up, training other staff members, app onboarding, familiarisation sessions with participants, use during 1–1 sessions and data export. 14 respondents reported not using the app themselves, however had seen it being used.

#### Overall views of the AR app

Most CAYA described the app's interface and navigation as “user friendly” and “simple.” Several CAYA commented positively on specific app features. Two noted that the RPE scale was a welcome addition, appreciating its use of colours and emojis. One CAYA reported that the exercise diary was helpful for tracking progress, while another expressed approval of the “skip” function, which allowed greater flexibility in navigating the exercise sessions.

68% of exercise professionals (13/19) who had gone through the onboarding process found it easy/very easy and all of those who had conducted familiarisation sessions (*n* = 15) reported finding it easy/very easy. Exercise professionals consistently described the app as “self-explanatory” and “easy to set up”. However, most reported that practical demonstrations during the familiarisation session were more effective than verbal explanations alone. Exercise professionals also recognised the value of the exercise diary, noting its potential to support adherence and monitoring. However, some exercise professionals noted that resetting the app to modify an individualised exercise programme removed access to previously logged exercise entries. Similarly, CAYA sessions were not recorded unless the whole session was completed.

CAYA and exercise professionals suggestions for further development included incorporating gamification features such as earning points or coins, introducing levels and rewards, and enabling users to set personalised goals. Exercise professionals also proposed linking rewards (e.g., coins or points) to avatar customisation.

#### Portability of the device/remote training

CAYA provided largely positive feedback regarding the use of a mobile device they could take home. Younger participants particularly noted enjoying the opportunity to use the app collaboratively with siblings at home. Among teenage participants, several reported that the app facilitated independent exercise and enhanced motivation to engage in physical activity. Some within this teenage sample also expressed that the individualised nature of the app increased their confidence in the safety of the prescribed exercises. In contrast, a few CAYA reported barriers to use at home, citing lack of time, preference for alternative activities, or feeling too unwell to engage with the app.

Exercise professionals also reported that the app served as an effective remote tool to support and motivate unsupervised exercise. They emphasised that its transportable nature enabled CAYA to complete exercises in a variety of settings. While only 40% (6/15) of exercise professionals who used the app within one-to-one sessions found it useful/very useful, they did acknowledge its potential as a valuable supplementary tool, instead of a substitute for in person sessions.

#### Exercises

There was mostly positive feedback in relation to the exercises providing “‘a good level of intensity’” and “‘pace’”, with “‘good variety’”. However, some CAYA and exercise professionals reported that the exercises were “'too easy and short’” for some users. Exercise professionals widely valued the app's capacity to individualise exercises for patients. Many reported that the range of available exercises was appropriate, and they appreciated the inclusion of exercises targeting the lower body, upper body, and core, along with seated versions to support accessibility for CAYA with specific physical needs. There was consistent feedback advocating for further individualising prescriptions, particularly with respect to adjusting repetitions and sets beyond the pre-set parameters. In addition, exercise professionals recommended incorporating further instructions within the app, such as the use of weights, to support progression.

#### AR technology

Perspectives varied regarding the AR technology used within the app. Several CAYA reported that they enjoyed the novel approach to physical activity facilitated by AR, describing the technology as “interesting” and “clever”. Some noted that the AR-based demonstrations were preferable to traditional instruction formats such as videos or written instructions. For example, CAYA highlighted the usefulness of being able to move around and view the AR avatar from multiple angles. Across all age groups, participants frequently reported the AR demonstrations as “helpful” when the technology functioned as intended, particularly when the avatar appeared promptly and in an appropriate location. One participant said it was nice seeing the avatar appearing in a familiar location. Among younger participants, some described the experience of locating the avatar in their physical environment as “fun” or “funny,” suggesting that the interactive element held some appeal.

Conversely, two CAYA expressed reservations about the use of AR. One indicated a general dislike for using technology to be active, while another stated that standard 2D video formats would be easier to follow. Technical issues were a recurring theme across all age groups, and by exercise professionals. Common concerns included the avatar “not appearing,” “not appearing quickly”, or “appearing in an odd location”. CAYA frequently described these experiences as “annoying” and “demotivating.” There were reports from CAYA and exercise professionals that the size of the room, and lighting seemed to affect the placement of the avatar within the environment which further impacted its usability. Additionally, some CAYA and exercise professionals reported finding the requirement to scan their surroundings between each exercise annoying.

#### Avatar design

CAYA generally responded positively to the avatar design, frequently describing it as “good” or “cool.” There was, however, consistent feedback indicating a desire for greater avatar customisation. One participant expressed a preference for a more human-like avatar, while participants across all age ranges, and exercise professionals, recommended offering a wider selection of avatar types, including animals and superheroes. Additionally, one participant noted that the avatar's skin tone differed from their own, and several CAYA and exercise professionals suggested the option to personalise the avatar to resemble the user more closely and thus enhance user engagement.

#### Suggested improvements and views on future use

Specific suggestions for improvement to the app, provided by both exercise professionals and CAYA are presented in Table 2. Exercise professionals also provided their views on future use. 90% (28/31) exercise professionals said they would recommend the AR app to other childhood cancer centres, especially as a supplementary tool to support an existing in person programme. One exercise professional suggested that the app would be particularly helpful for isolated patients and another reflected that the app may be useful for “other chronically ill children”. There were mixed views regarding the most appropriate target age range for the current version of the app. While some felt it could be suitable for children as young as four years, there was general consensus that the app may be most suitable for children aged approximately 8–14 years. Exercise professionals expressed that the current basic interface and child-like avatar design lends itself to younger users, while older users might benefit from additional features, such as a more sophisticated interface or integrated social functionalities, or might require a different app altogether. Moreover, some reported that parents expressed concerns about introducing additional technology into their child's daily routine.

**Table 2:** Suggested recommendations for app improvement.

Category	Recommendation	Reported by	Example quotes
AR	i) AR technology improved to ensure quick and consistent appearance	CAYA and exercise professionals	“Additionally, I think the AR technology within the application needs to be significantly improved as the avatar not appearing quickly was the most frequent problem reported” *(*exercise professional*)* “It was sometimes difficult to make the avatar appear.” *(CAYA, age 11 years)*
Avatar	i) Additional avatar options (superheroes/animals)	CAYA and exercise professionals	“It would be nice to have other avatar options, for example animals or superheroes.” *(CAYA, age 17 years)* “I think there should be more options for other styles of avatars to engage more children with different interests, such as superheroes or cartoons.” *(*exercise professional)
	ii) Avatar customisation	CAYA and exercise professionals	“I liked the avatar but I think it would be better if you could customise it so it could look like you.” *(CAYA, age 12 years)* “The avatar [should be] customised by each patient” *(*exercise professional*)*
Exercise prescription	i) Further flexibility when selecting sets/reps	Exercise professionals	“it would be helpful, if you can decide for each session how many reps and sets should be performed” *(*exercise professional*)*
	ii) Additional exercise options	CAYA and exercise professionals	“More stretches in the app.” *(CAYA, age 16 years)* “I think that more exercises could be added to the exercise catalogue, to enable greater variety for the app users.” *(*exercise professional*)* “It would also be nice to include other exercises including more stretches or balance exercises” *(*exercise professional*)*
Exercise diary	i) Diary saving workouts regardless of completion	Exercise professionals	“the programming needs to be changed so that the exercise session is recorded regardless of whether the participant completed the whole workout” *(*exercise professional*)* “There should be an option on each exercise to ‘finish now’ and the app record how many exercises were completed. Often we would receive feedback that a child did the exercise but because they did not get to the very end it was not recorded.” *(*exercise professional*)*
	ii) Diary data saved when adjustments are made to the exercise prescription	Exercise professionals	“[..] This then required the app to be reset to change the exercise prescription. Unfortunately this meant that the exercise diary was cleared and the participant could no longer see their previous workouts. It would be a nice option if you were able to edit their exercise programme rather than restart the app all together” *(*exercise professional*)*
Gamification	i) Challenges	Exercise professionals	“Also some more playful content (challenges [..] might be helpful.” *(*exercise professional*)* “But I think that the APP is target for young (8–12) and so it needs more game and challenges (like: if you do this training you earn a pair of shoes)” *(*exercise professional*)*
	ii) Levels	CAYA	“It would be great to have different levels, so it feels more like a game and you can reach new levels and get better by this.” *(CAYA, age 16 years)*
	iii) Rewards (e.g., points or coins)	CAYA and exercise professionals	“It would be good to collect coins or points to earn things for your avatar, for example clothes” *(CAYA, age 17 years)* “[..] it would be a nice addition to include a reward system whereby participants receive coins or points which allow them to customise their avatar for example. *(*exercise professional*)*
	iv) Usage streaks	CAYA	“It would be good if there was some audio feedback and a streak function to motivate you to use it.” *(CAYA, age 11 years)*
	v) Goal setting	CAYA	“It would be great if you can set goals” *(CAYA, age 16 years)*

CAYA: children, adolescents and young adults with cancer.

## Discussion

As physical activity and exercise plays a crucial role in ameliorating the physical and psychological side effects of cancer and its treatment (21), there is an increased interest in mHealth apps that support engagement in safe and accessible exercise interventions for CAYA with cancer (22). This is the first AR exercise app that has been developed specifically for children and young people with cancer. Our findings contribute to this growing field by providing insights into perspectives of AR technologies. Importantly, the study highlights both the potential and the challenges of integrating such tools into existing in-person exercise programmes in oncology care, from the dual perspectives of CAYA and exercise professionals.

Both CAYA and exercise professionals reported generally positive experiences of the app, reporting the AR demonstrations as novel, engaging and helpful. The portability of the smartphone was also viewed favourably by both CAYA and exercise professionals as it supported exercise programmes outside of the clinical setting. However, there was strong consensus that the app should serve to supplement and not replace face to face sessions. Despite many positive comments, technical limitations were also noted. There were comments that the AR technology did not always function reliably, and that the exercise diary required further development. Recommendations for further development included the addition of gamification elements, and the customisability of avatars.

### The use of apps to support exercise programmes in paediatric oncology

Both CAYA and exercise professionals valued the flexibility of being able to use the app at home. This highlights how the integration of mobile apps into paediatric oncology exercise programmes offers a promising way to support and enhance engagement and continuity of care beyond clinical environments (22). It also aligns with previous research demonstrating the potential of digital health technology to mitigate barriers to access, especially for families living in rural or underserved areas (23). However, the financial and equity implications of implementing such technology outside of a research setting must be considered. In this study, CAYA were provided with a study smartphone and the necessary accessories, but this may not be feasible in routine clinical practice. The costs of devices, maintenance and technical support could represent a barrier for some healthcare systems and families, potentially exacerbating existing inequalities in access to supportive exercise interventions. Therefore, future implementation of AR-supported exercise programmes should prioritise equitable access, for example by ensuring compatibility with personal devices, offering institutional device lending schemes, or integrating them within existing digital health infrastructures.

It is equally important to consider concerns regarding digital tools in paediatric populations. These concerns are not limited to the healthcare context but rather reflect a broader societal apprehension surrounding the rise in excessive screen time in children and growing dependence on digital devices for entertainment and education (24). In the context of this work exercise professionals reported instances where parents were hesitant or avoided using the AR app due to these concerns. In addition to screen time, other research has highlighted parental concerns about data protection and the wider suitability of app content, including advertisements (25). While such issues are typically well-regulated within controlled research environments, the broader dissemination of mHealth tools must include safeguards to protect users.

Previous research has also identified concerns among parents of children with complex needs regarding the potential for such interventions to replace, rather than complement, essential supportive services (26). Notably, exercise professionals in this study emphasised that the AR app should serve as an adjunct to, rather than substitute for in person care. These findings underscore the importance of integrating digital tools in a way that enhances, rather than compromises, the delivery of personalised care in paediatric oncology.

### Usability and technical considerations of AR apps

Within this study, CAYA and exercise professionals appreciated the added benefits of the AR components, however both noted frustrations with technical issues. Usability challenges, such as problems with software design, interface, or content are well documented barriers to app adoption and continued use (27, 28). While AR technology appeared to offer added value over conventional exercise instructions methods (i.e., written or 2D videos) for many users, not all CAYA perceived it as beneficial. This highlights the importance of balancing technological novelty with usability, as well as the challenge of addressing diverse user needs and preferences within a single app. To optimise the effectiveness of AR-enhanced mHealth tools, developers should focus on ensuring reliability of the AR features, while also considering offering alternative formats to accommodate diverse user preferences.

### Tracking engagement and adherence through app-based features

The exercise diary was intended to track usage patterns of the app and provide CAYA with the opportunity to monitor their own engagement with the app. Feedback and monitoring for users to track their performance and status are important factors to influence trust, motivation and engagement (25, 29). Incorporation of such features could support processes such as goal achievement, helping young people to enhance their physical fitness levels through self-monitoring, self-motivation, and self- surveillance (30). However, exercise professionals highlighted that there was sometimes a discrepancy between what CAYA verbally reported compared to what was recorded in the app exercise diary, with regards to completing exercise sessions. This variation raises important questions about user engagement and adherence to the prescribed programme. Additional feedback pointed at other potential pitfalls within the app design, as the exercise session had to be completed in order for the session to be recorded in the exercise diary. These findings highlight the need for more robust monitoring and feedback mechanisms within app-based exercise interventions. Features such as semi-automated tracking may be a favourable addition to support awareness in behaviour and engagement, while reducing the burden of fully manual tracking and accuracy challenges of fully automated tracking (31).

### Enhancing engagement through personalisation and gamification

A key theme identified by both CAYA and exercise professionals was the potential to enhance user engagement through increased interactivity, personalisation and gamification within the app. Personalisation features, such as customising avatars, have been successfully implemented in other apps for childhood cancer patients (32). In their study, Fortier and colleagues (2016) allowed users to earn virtual coins through app engagement to purchase accessories and modify interface design. Evidence also suggests that avatars resembling the user can foster a stronger sense of identification and promote healthier behaviours compared to generic avatars (33). In parallel, CAYA and exercise professionals highlighted the value of gamified elements such as challenges, levels, and rewards to sustain interest and motivation. This aligns with wider trends in mHealth and eHealth, where gamification has been shown to support goal setting, provide positive reinforcement, and encourage long-term use (34). In paediatric oncology, where children face additional barriers to exercise (35), such features could be especially effective in boosting motivation and adherence. However, it is essential for these elements to be adaptable to the diverse needs and preferences of this population.

### Strengths and limitations

A key strength of this study is its large, multi-centre design across multiple countries across Europe. By including a diverse range of clinical sites, the study enhances the generalisability of its findings to a broader European context with different clinical settings, staffing and resources. Secondly, this study is among the first to qualitatively explore the perspectives of both children and young people undergoing treatment for cancer, and exercise professionals, on the use of an AR exercise app. By including feedback of end users and professionals, this study provides a holistic understanding of the usability and perspectives of implementing an AR app to support an individualised exercise programmes. Finally, the study contributes to an emerging area of research in the field of mHealth within paediatric oncology care, highlighting the potential and limitations of AR tools. The inclusion of feedback of both functionality and user experience enables practical recommendations for further technology development.

The evaluation of the AR app was based on a sub-study of a larger randomised clinical trial. As such, only a small subset (approx. 10%) of the total RCT participants took part in the semi-structured interviews evaluating the AR app. This may be explained by the fact that only selected study sites participated in the AR app subproject. Moreover, additional eligibility criteria (e.g., age restrictions) were applied, and the duration of the subproject was shorter than that of the overall RCT. Additionally, the number of study iPhones available at participating centres was limited, which may have contributed to the proportion of participants who were not offered access to the app. Furthermore, it is possible that the interview responses were affected by confirmation or social desirability bias and the presence of a parent/guardian may have also affected participant responses. Interviews were not audio-recorded and were conducted in the native language of each centre and translated into English, which may have resulted in some loss of nuance. Exercise professional feedback was collected via an online form in English and depending on the fluency of the respondents, some information may have been lost or misreported.

## Conclusion

This study highlights the potential of novel AR technology to enhance engagement in exercise among children and young people undergoing cancer treatment. CAYA and exercise professionals reported the value of features including visual exercise demonstrations, and the opportunity to continue the exercise programme during home stays or away from a clinical setting. Feedback emphasised the importance of technological reliability with regards to the AR components, and suggested improvements, such as a customisable avatar, and gamified features. While AR offers promising benefits within paediatric oncology care, it must be integrated in a way to support and not replace face-to-face exercise interventions. Future development should focus on user-centred design, increased individualisation, and equity of access for CAYA with cancer and their families. Additionally further research is needed to evaluate long-term engagement and clinical outcomes.

## Data Availability

The datasets presented in this article are not readily available because the data will be available from the corresponding author upon reasonable request. Requests to access the datasets should be directed to Eila Watson - ewatson@brookes.ac.uk.

## Appendix A. FORTEe sub-project recruiting centres:

Oxford University Hospitals Foundation NHS Trust in collaboration with Oxford Brookes University (UK), Childhood Cancer Center of the University Medical Center of the Johannes Gutenberg-University Mainz (Germany) & Heidelberg University Hospital (Germany), Fondazione Monza e Brianza per Il Bambino e La Sua Mamma, Monza (Italy) & IRCCS National Cancer Institute Foundation (Italy)

## Appendix B. Interview questions with prompts

Question 1 - What did you like about the AR app?

Question 2 - What did you not like about the AR app?

Interview topic prompts:

- Avatar design
- Exercise demonstrations
- AR technology [avatar (not) appearing]
- App interface (design, colours, font)
- What kind of avatar would be preferred
- Equipment (iPhone, tripod, accessories provided)
- The amount of exercises in the workouts (too intense or easy?)
- The types of exercises in the workouts
- The exercise diary (being able to check their workout history)
- Being able to take the phone home

## References

[1] Klimczak KS Levin ME. Mobile health. In: Friedman-Wheeler DG Wenzel A, editors. The Sage Encyclopedia of Mood and Anxiety Disorders. Thousand Oaks, CA: SAGE Publications, Inc. (2025). p. 783–7. 10.4135/9781071886229.n278

[2] Gentili A Failla G Melnyk A Puleo V Tanna GLD Ricciardi W The cost-effectiveness of digital health interventions: a systematic review of the literature. Front Public Health. (2022) 10:787135. 10.3389/fpubh.2022.787135 36033812 PMC9403754

[3] Edwards J Waite-Jones J Schwarz T Swallow V. Digital technologies for children and parents sharing self-management in childhood chronic or long-term conditions: a scoping review. Children (Basel). (2021) 8:1203. 10.3390/children8121203 34943399 PMC8700031

[4] Cheung AT Li WHC Ho LLK Ho KY Chan GCF Chung JOK. Physical activity for pediatric cancer survivors: a systematic review of randomized controlled trials. J Cancer Surviv. (2021) 15:876–89. 10.1007/s11764-020-00981-w 33389553 PMC7778568

[5] Ramsey WA Heidelberg RE Gilbert AM Heneghan MB Badawy SM Alberts NM. Ehealth and mHealth interventions in pediatric cancer: a systematic review of interventions across the cancer continuum. Psychooncology. (2020) 29:17–37. 10.1002/pon.5280 31692183

[6] Jibb LA Cafazzo JA Nathan PC Seto E Stevens BJ Nguyen C Development of a mHealth real-time pain self-management app for adolescents with cancer: an iterative usability testing study [formula: see text]. J Pediatr Oncol Nurs. (2017) 34:283–94. 10.1177/1043454217697022 28376666

[7] Vaughn J Shah N Jonassaint J Harris N Docherty S Shaw R. User-centered app design for acutely ill children and adolescents. J Pediatr Oncol Nurs. (2020) 37:359–67. 10.1177/1043454220938341 32646317 PMC7802024

[8] Kim Y Seo J An S-Y Sinn DH Hwang JH. Efficacy and safety of an mHealth app and wearable device in physical performance for patients with hepatocellular carcinoma: development and usability study. JMIR Mhealth Uhealth. (2020) 8:e14435. 10.2196/14435 32159517 PMC7097723

[9] Groarke JM Richmond J Mc Sharry J Groarke A Harney OM Kelly MG Acceptability of a mobile health behavior change intervention for cancer survivors with obesity or overweight: nested mixed methods study within a randomized controlled trial. JMIR Mhealth Uhealth. (2021) 9:e18288. 10.2196/18288 33591290 PMC7925146

[10] Berg CJ Stratton E Esiashvili N Mertens A. Young adult cancer survivors’ experience with cancer treatment and follow-up care and perceptions of barriers to engaging in recommended care. J Cancer Educ. (2016) 31:430–42. 10.1007/s13187-015-0853-9 25948413 PMC4712118

[11] Roy I Salles J Neveu E Lariviére-Bastien D Blondin A Levac D Exploring the perspectives of health care professionals on digital health technologies in pediatric care and rehabilitation. J Neuroeng Rehabil. (2024) 21:156. 10.1186/s12984-024-01431-9 39261920 PMC11391714

[12] Iqbal AI Aamir A Hammad A Hafsa H Basit A Oduoye MO Immersive technologies in healthcare: an in-depth exploration of virtual reality and augmented reality in enhancing patient care, medical education, and training paradigms. J Prim Care Community Health. (2024) 15:21501319241293311. 10.1177/21501319241293311 39439304 PMC11528804

[13] Mendoza-Ramírez CE Tudon-Martinez JC Félix-Herrán LC Lozoya-Santos JdJ Vargas-Martínez A. Augmented reality: survey. Appl Sci. (2023) 13:10491. 10.3390/app131810491

[14] Liszio S Bäuerlein F Hildebrand J van Nahl C Masuch M Basu O. Cooperative virtual reality gaming for anxiety and pain reduction in pediatric patients and their caregivers during painful medical procedures: protocol for a randomized controlled trial. JMIR Res Protoc. (2025) 14:e63098. 10.2196/63098 40164171 PMC11997540

[15] Al-Ansi AM Jaboob M Garad A Al-Ansi A. Analyzing augmented reality (AR) and virtual reality (VR) recent development in education. Soc Sci Humanit Open. (2023) 8:100532. 10.1016/j.ssaho.2023.100532

[16] Oh H Son W. Cybersickness and its severity arising from virtual reality content: a comprehensive study. Sensors (Basel). (2022) 22:1314. 10.3390/s22041314 35214216 PMC8963115

[17] Straun K Marriott H Solera-Sanchez A Windsor S Neu MA Dreismickenbecker E The development of an augmented reality application for exercise prescription within paediatric oncology: app design and protocol of a pilot study. Health Informatics J. (2024) 30:14604582241288784. 10.1177/14604582241288784 39447216

[18] Neu MA Dreismickenbecker E Lanfranconi F Stössel S Balduzzi A Wright P Get strong to fight childhood cancer - an exercise intervention for children and adolescents undergoing anti-cancer treatment (FORTEe): rationale and design of a randomized controlled exercise trial. BMC Cancer. (2025) 25:1275. 10.1186/s12885-025-14489-y 40775303 PMC12330123

[19] Kuckartz U. Qualitative Text Analysis: A Guide to Methods, Practice & Using Software (2014). Available online at: https://www.academia.edu/89311675/Qualitative_Text_Analysis_A_Guide_to_Methods_Practice_and_Using_Software (Accessed July 10, 2025).

[20] Mayring P. Qualitative content analysis. Forum Qual Soc Res. (2000) 1(2):Art. 20. 10.17169/fqs-1.2.1089

[21] Morales JS Valenzuela PL Velázquez-Díaz D Castillo-García A Jiménez-Pavón D Lucia A Exercise and childhood cancer—a historical review. Cancers (Basel). (2022) 14:82. 10.3390/cancers14010082 PMC8750946 35008246

[22] Skeens MA Jackson DI Sutherland-Foggio MS Sezgin E. Mhealth apps in the digital marketplace for pediatric patients with cancer: systematic search and analysis. JMIR Pediatr Parent. (2024) 7:e58101. 10.2196/58101 39352720 PMC11460307

[23] Ha L Nevin SM Wakefield CE Jacovou J Mizrahi D Signorelli C. Exploring childhood cancer survivor, parent, healthcare and community professionals’ experiences of, and priorities for, using digital health to engage in physical activity: a mixed methods study. J Cancer Surviv. (2024) 19:1403–18. 10.1007/s11764-024-01560-z 38478196 PMC12283819

[24] Radesky JS Schumacher J Zuckerman B. Mobile and interactive media use by young children: the good, the bad, and the unknown. Pediatrics. (2015) 135:1–3. 10.1542/peds.2014-2251 25548323

[25] Lee J Su Z Chen Y. Mobile apps for children’s health and wellbeing: design features and future opportunities. AMIA Annu Symp Proc. (2024) 2023:1027–36. PMID: 38222362 38222362 PMC10785842

[26] Apps J Webb S Hutton E. Parents’ and carers’ attitudes to the use of digital technology and its role in the care of children with complex needs. Br J Occup Ther. (2024) 87:452–60. 10.1177/03080226241233112 40336719 PMC11887867

[27] Giebel GD Speckemeier C Abels C Plescher F Börchers K Wasem J Problems and barriers related to the use of digital health applications: scoping review. J Med Internet Res. (2023) 25:e43808. 10.2196/43808 37171838 PMC10221513

[28] Ajit G. A systematic review of augmented reality in STEM education. Stud Appl Econ. (2021) 39:1–19. 10.25115/eea.v39i1.4280

[29] Wang J-W Zhu Z Shuling Z Fan J Jin Y Gao Z-L Effectiveness of mHealth app–based interventions for increasing physical activity and improving physical fitness in children and adolescents: systematic review and meta-analysis. JMIR Mhealth Uhealth. (2024) 12:e51478. 10.2196/51478 38687568 PMC11094610

[30] Yang J Casey A Cale L. The influence of healthy lifestyle technologies on young people’s physical activity participation and health learning: a systematic review. Quest. (2024) 76:72–92. 10.1080/00336297.2023.2218038

[31] Choe EK Abdullah S Rabbi M Thomaz E Epstein DA Cordeiro F Semi-Automated tracking: a balanced approach for self-monitoring applications. IEEE Pervasive Comput. (2017) 16:74–84. 10.1109/MPRV.2017.18

[32] Fortier MA Chung WW Martinez A Gago-Masague S Sender L. Pain buddy: a novel use of m-health in the management of children’s cancer pain. Comput Biol Med. (2016) 76:202–14. 10.1016/j.compbiomed.2016.07.012 27479493 PMC5639256

[33] Franco M Monfort C Piñas-Mesa A Rincon E. Could avatar therapy enhance mental health in chronic patients? A systematic review. Electronics (Basel). (2021) 10:2212. 10.3390/electronics10182212

[34] Willinger L Schweizer F Böhm B Scheller DA Jonas S Oberhoffer-Fritz R Evaluation of the gamified application KIJANI to promote physical activity in children and adolescents: a multimethod study. Digital health. (2024) 10:20552076241271861. 10.1177/20552076241271861 39161345 PMC11331568

[35] Ross WL Le A Zheng DJ Mitchell H-R Rotatori J Li F Physical activity barriers, preferences, and beliefs in childhood cancer patients. Support Care Cancer. (2018) 26:2177–84. 10.1007/s00520-017-4041-9 29383508

